# Increase the Content of Ester Compounds in Blueberry Wine Fermentation with the Ester-Producing Yeast: *Candida glabrata*, *Pichia anomala*, and *Wickerhamomyces anomalus*

**DOI:** 10.3390/foods11223655

**Published:** 2022-11-16

**Authors:** Wenqin Cai, Bang Li, Yanru Chen, Guiming Fu, Haowei Fan, Mengfei Deng, Yin Wan, Na Liu, Mengxiang Li

**Affiliations:** 1 State Key Laboratory of Food Science and Technology, College of Food Science and Technology, Nanchang University, Nanchang 330047, China; 2 International Institute of Food Innovation, Nanchang University, Nanchang 330299, China

**Keywords:** ester-producing yeasts, co-fermentation, *Saccharomyces cerevisiae*, ester compounds

## Abstract

The co-fermentation of *Saccharomyces cerevisiae* and ester-producing yeasts is considered to be an effective way to improve the flavor and quality of fruit wine. In this study, three kinds of ester-producing yeasts (*Candida glabrata* NCUF308.1, *Pichia anomala* NCUF306.1, and *Wickerhamomyces anomalus* NCUF307.1) and *S. cerevisiae* NCUF309.2 were used to simulate blueberry wine co-fermentation at different ratios. The results showed that, compared with *S. cerevisiae* NCUF309.2 fermentation (S), the population of *S. cerevisiae* NCUF309.2 in co-fermentation samples decreased to varying degrees, and the content of ethanol also decreased. The results also showed that the co-fermentation of *C. glabrata* NCUF308.1 and *S. cerevisiae* NCUF309.2 at the ratio of 1:1 (CS1), co-fermentation of *P. anomala* NCUF306.1 and *S. cerevisiae* NCUF309.2 at the ratio of 5:1 (PS5), and co-fermentation of *W. anomalus* NCUF307.1 and *S. cerevisiae* NCUF309.2 at the ratio of 5:1 (WS5) could significantly increase the content of ester compounds (*p* < 0.05), which was 3.29, 4.75, and 3.04 times that of the S sample, respectively. Among them, the sample of CS1 was characterized by phenethyl acetate and isoamyl acetate, while the samples of CS5 and PS5 were characterized by propyl octanoate and ethyl decanoate, and the sample of WS5 was characterized by 3-methylbutyl hexanoate. However, the contents of odor active compounds were higher in the CS1 sample. Therefore, the samples of CS1 had the potential to create the distinctive flavor of blueberry wine.

## 1. Introduction

Blueberries are known to taste both sour and sweet and have a mellow bouquet. Upon consumption, blueberries can not only supplement various vitamins and trace elements, but also enhance immune function and anti-aging, especially having an obvious protective effect on cardiovascular disease and renal failure [1]. However, blueberries are extremely susceptible to decay during storage and processing; thus, blueberry wine provides an alternative method to preserve nutrition and extend sales time [2,3]. However, the current strain used for blueberry wine is generally just *Saccharomyces cerevisiae*, which makes the flavor of blueberry wine relatively simple and cannot meet the demand of current consumers for blueberry wine with various flavors.

At present, ester-producing yeasts received significant attention for their role in both ethanol yield reduction and flavor enhancement in wine [4,5]. Studies found that ester-producing yeasts, belonging to the genera of *Hanseniaspora*, *Candida*, *Metschnikowia*, *Pichia*, *Wickeramomyces*, and *Torulaspora*, had some desired oenological features that were absent in *S. cerevisiae*, which could make up for the deficiency of pure fermentation of *S. cerevisiae* [6,7,8,9,10]. Ester-producing yeasts can change the fermentation environment, flavor, and quality of wine in two ways. One way is that ester-producing yeasts can catalyze the release of volatile aroma compounds from non-volatile precursors [11]. Studies showed that ester-producing yeasts could secret enzymes (esterase, β-glycosidase, lipase, and protease) that hydrolyze glycosylated odorless precursors in grapes to form free volatile aroma compounds, thus improving the flavor of grape wine [12,13,14]. Swangkeaw et al. found that high concentration of volatile aroma compounds was detected in wine treated by β-glucosidases from *Pichia anomala* [15]. Another way is that ester-producing yeasts can directly produce volatile aroma compounds through their metabolic activities [16]. For example, *Williopsis saturnus*, recently renamed *Cyberlindnera saturnus*, could produce a large number of ester compounds (phenylethyl acetate and isoamyl acetate) in cider fermentation [17]. Ye et al. also improved the quality of apple cider by co-fermentation of *Wickerhamomyces anomalus* and *S. cerevisiae* [18]. The mixed fermentation of *P. anomala* and *S. cerevisiae* could also improve the sensory quality of cane wine because their co-fermentation increased the volatile compounds that were associated with a good sensory description [19]. Among them, ester compounds formed by the reaction of alcohol and carboxylic acids are vital volatile aroma compounds that contribute to the flavor characteristics of fruit wine [20,21]. Therefore, the co-fermentation of ester-producing yeasts and *S. cerevisiae* is an attractive strategy to increase the content of ester compounds, thus improving the flavor of blueberry wine [22].

In our previous study, ester-producing yeasts (*Candida glabrata* NCUF308.1 (E4), *P. anomala* NCUF306.1 (E1), and *W. anomalus* NCUF307.1 (E3)) with strong abilities of ester production were selected from blueberry peel and fermented *Baijiu Jiupei* [23]. Therefore, three kinds of ester-producing yeasts and *S. cerevisiae* NCUF309.2 were used to simulate the co-fermentation of blueberry wine under different inoculation ratios, respectively. The growth of yeasts, fermentation characteristics, and the accumulation of ester compounds during the fermentation were studied to obtain the optimal ratio of *S. cerevisiae* and ester-producing yeast for the co-fermentation of blueberry wine. Our results lay the foundation for the further analysis of the application of ester-producing yeasts and *S. cerevisiae* in blueberry wine.

## 2. Materials and Methods

### 2.1. Strains and Media

*S. cerevisiae* NCUF309.2, *P. anomala* NCUF306.1, *W. anomalus* NCUF307.1, and *C. glabrata* 308.1 were cultured in yeast extract peptone dextrose (YPD) medium (peptone 20 g/L, yeast extract powder 10 g/L, glucose 20 g/L) at 180 rpm, and 28 °C to logarithmic phase.

Synthetic medium used to simulate the fermentation of blueberry wine was prepared based on the method described by Rossouw et al. [24]. The ingredients of the synthetic medium were as follows: 100 g/L glucose, 100 g/L fructose, 1.5 g/L yeast extract, 0.3 g/L citric acid, 5 g/L tartaric acid, 5 g/L malic acid, 2 g/L ammonium sulfate, 5 g/L potassium dihydrogen phosphate, 0.5 g/L magnesium sulfate, 0.2 g/L sodium chloride, and 0.05 g/L manganese sulfate. The pH of the medium was adjusted to 4.0, with 0.5 M sodium hydroxide. Then, the medium was sterilized at 121 °C for 20 min.

### 2.2. Fermentation Conditions

**Figure 1:**
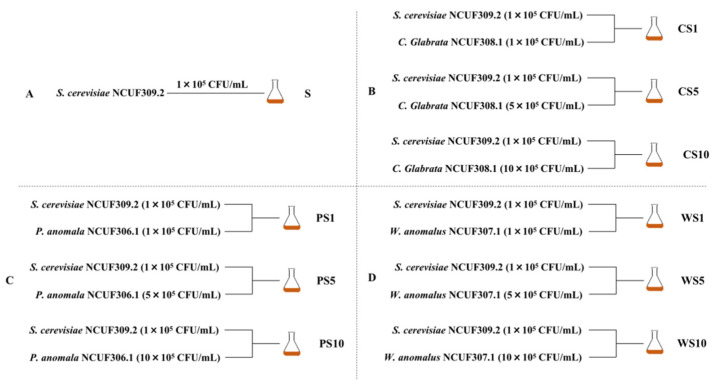
Fermentation conditions of pure fermentation of *S. cerevisiae* NCUF309.2 and co-fermentation of ester-producing yeasts and *S. cerevisiae* NCUF309.2 under different ratios. (**A**) pure fermentation of *S. cerevisiae* NCUF309.2; (**B**) co-fermentation of *C. glabrata* NCUF308.1 and *S. cerevisiae* NCUF309.2; (**C**) co-fermentation of *P. anomala* NCUF306.1 and *S. cerevisiae* NCUF309.2; (**D**) co-fermentation of *W. anomalus* NCUF307.1 and *S. cerevisiae* NCUF309.2.

The co-fermentation model of three ester-producing yeasts and *S. cerevisiae* NCUF309.2 at ratios of 1:1, 5:1, and 10:1 is shown in Figure 1. Anyway, *S. cerevisiae* NCUF309.2 inoculated with 1 × 10^5^ CFU /mL for pure fermentation was used as control (Figure 1A), and all of them were fermented at 26 °C for 7 d under static conditions.

### 2.3. Determination of Growth of Yeasts

Samples were taken every 24 h during the simulated fermentation of blueberry wine and diluted onto Wallerstein laboratory nutrient (WLN) medium (Hopebiol, Qingdao, China), then incubated at 28 °C for 2 d. Different yeast colonies on the WLN plates presented different morphologies and colors. Therefore, colonies on the WLN plate were counted according to the differences in colony morphologies, and the yeast populations were expressed as CFU/mL.

### 2.4. Determination of the Content of Reducing Sugar and Ethanol

Samples were taken every 24 h during the simulated fermentation of blueberry wine, and the contents of ethanol and reducing sugar were determined via alcoholmeter after distillation and Fehling’s solution, according to the method of Yin et al. [25]. The contents of reducing sugar and ethanol were expressed as g/L and vol%, respectively.

### 2.5. Analysis of Ester Compounds

The ester compounds were analyzed by headspace solid-phase microextraction–gas chromatography–mass spectrometry (HS-SPME–GC–MS). A total of 10 mL sample was placed into a 20 mL headspace bottle, followed by the addition of 1.0 g sodium chloride and 10 μL 4-methyl-2-pentanol as internal standard with 10 μg/L. Then, the mixture was placed in a water bath at 45 °C for 30 min. Meanwhile, the ester compounds were collected by a divinyl-benzene carboxen on polydimethylsiloxane-coated (DVB/CAR/PDMS) fiber (Supelco Co., Bellefonte, PA, USA) for 40 min. After extraction, the fiber was inserted into the injection port of the gas chromatography–mass spectrometry (GC–MS) for desorption at 250 °C for 5 min. 

The extracts were analyzed using GC–MS (Agilent Technologies 8860/5977B, Palo Alto, CA, USA) equipped with an HP-5MS column (30 m × 0.25 mm × 0.25 μm, J & W Scientific, Folsom, CA, USA). The condition of HS-SPME–GC–MS was based on the method described by Shi et al., with a little modification [26]. The conditions of GC were as follows: 35 °C for 3 min at an increment of 3 °C/min to 160 °C and maintaining a constant temperature for 2 min, then 8 °C/min to 230 °C and maintaining for 3 min. The inlet temperature was set at 250 °C. The conditions of MS were as follows: the ion source was electron ionization (EI), the ion source temperature was 250 °C, electron impact ionization mode was at 70 eV, the scanning range was 33–450 *m*/*z*, and the scanning mode was full scan. The qualitative and quantitative analyses were carried out by the method of Ulrich et al. [27].

### 2.6. Data Analysis

All the experiments were performed in triplicates. All the data were processed and analyzed by Excel 2013 and Origin 9.0. Any significant differences among variances were determined by the one-way analysis of variance (ANOVA), with Duncan test of the SPSS 19.0 software (*p* < 0.05, there was a significant difference between the data). Simultaneously, SIMCA software (Version 14.1) was used for principal component analysis (PCA), and R software (Version 3.6.1) was used for hierarchical cluster analysis (HCA) of ester compounds.

## 3. Results and Discussion

### 3.1. Growth of Yeasts during the Simulated Fermentation of Blueberry Wine

**Figure 2:**
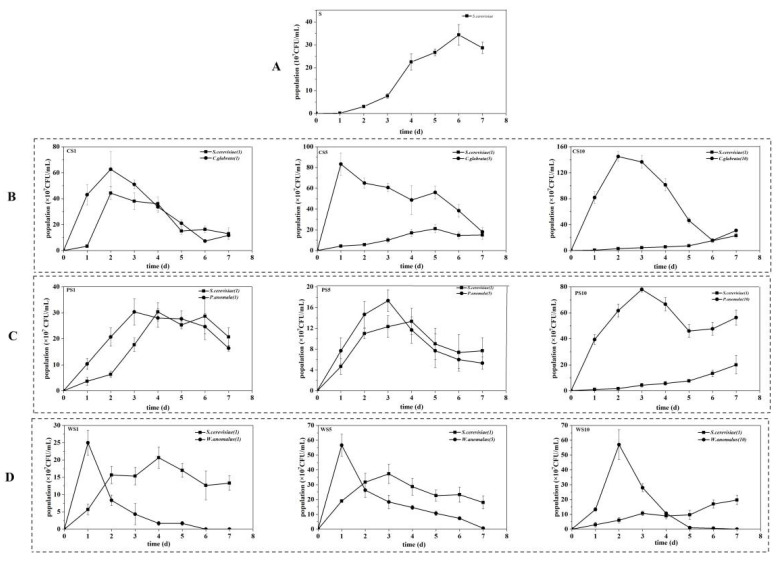
Growth of yeasts during the simulated fermentation of blueberry wine. (**A**) pure fermentation of *S. cerevisiae* NCUF309.2; (**B**) co-fermentation of *C. glabrata* NCUF308.1 and *S. cerevisiae* NCUF309.2; (**C**) co-fermentation of *P. anomala* NCUF306.1 and *S. cerevisiae* NCUF309.2; (**D**) co-fermentation of *W. anomalus* NCUF307.1 and *S. cerevisiae* NCUF309.2.

As presented in Figure 2A, the growth of *S. cerevisiae* NCUF309.2 in the S sample was slow in the first 3 d and rapid in 3–6 d, reaching the highest population of 3.43 × 10^8^ CFU/mL on 6 d. However, in the co-fermentation samples, ester-producing yeasts could adapt quickly to the fermentation environment and reach a growth peak in the first 3 d (Figure 2). Anyway, the addition of ester-producing yeasts could promote the growth of *S. cerevisiae* NCUF309.2 in the first 3 d, especially in the CS1, PS1, PS5, WS1, and WS5 samples (Table S1).

In the co-fermentation samples of CS, the growth of *S. cerevisiae* NCUF309.2 was obviously affected in the CS5 and CS10 samples (Figure 2B). Similarly, in the co-fermentation samples of PS (Figure 2C), the population of *S. cerevisiae* NCUF309.2 gradually decreased with the increase of *P. anomala* NCUF306.1 inoculation ratio. The reason for these phenomena was that ester-producing yeast competed with *S. cerevisiae* NCUF309.2 for nutrients, and the competition increased with the inoculation ratio of ester-producing yeast [28]. Anyway, we also found that the growth of *W. anomalus* NCUF307.1 was immediately inhibited by *S. cerevisiae* NCUF309.2, with a sharp decrease in population during fermentation. The growth inhibition phenomenon of ester-producing yeasts was also reported in other studies [29,30]. For example, even with a high inoculation of *Torulaspora delbrueckii* (twenty times more than *S. cerevisiae*) in wine fermentation, *S. cerevisiae* still had a negative impact on the viability of *T. delbrueckii* [31]. This could be attributed to cell–cell contact that induced the death of ester-producing yeasts [32]. 

These results indicated that different ester-producing yeasts and their inoculation ratios had different effects on the growth of *S. cerevisiae* NCUF309.2, thereby its ability to produce ethanol could be affected. Therefore, the fermentation characteristics during the simulated fermentation of blueberry wine were also studied.

### 3.2. The Content of Reducing Sugar and Ethanol during the Simulated Fermentation of Blueberry Wine

**Figure 3:**
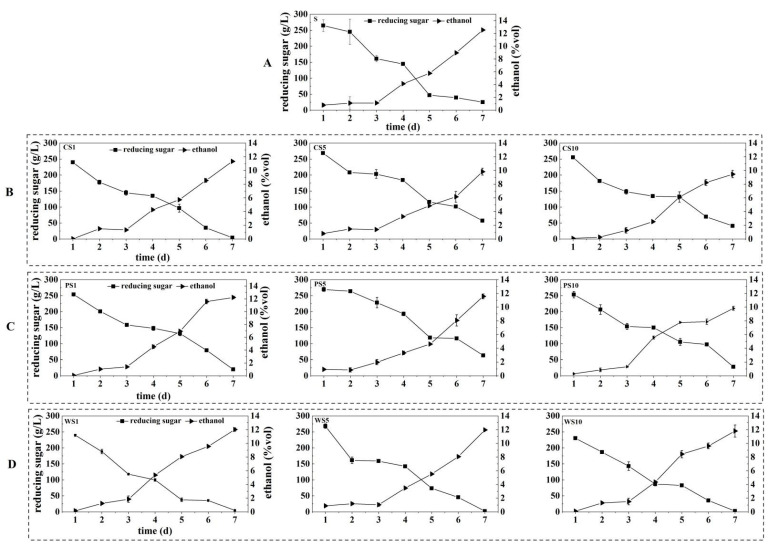
Changes in ethanol and reducing sugar contents of different ester-producing yeasts, with *S. cerevisiae* NCUF309.2 at different ratios during the simulated fermentation of blueberry wine. (**A**) pure fermentation of *S. cerevisiae* NCUF309.2; (**B**) co-fermentation of *C. glabrata* NCUF308.1 and *S. cerevisiae* NCUF309.2; (**C**) co-fermentation of *P. anomala* NCUF306.1 and *S. cerevisiae* NCUF309.2; (**D**) co-fermentation of *W. anomalus* NCUF307.1 and *S. cerevisiae* NCUF309.2.

As presented in Figure 3A, the content of reducing sugar in the S sample decreased rapidly during the first 5 d of fermentation, but the content of ethanol did not change too much during the first 3 d. After 3 d, the ethanol content increased sharply. Finally, the contents of reducing sugar and ethanol were 25.26 g/L and 12.52% (vol), respectively. These results indicated that the reducing sugar was mainly used for the growth of yeasts during the first 3 d of fermentation, and then was gradually converted to ethanol after 3 d [33]. 

**Figure 4:**
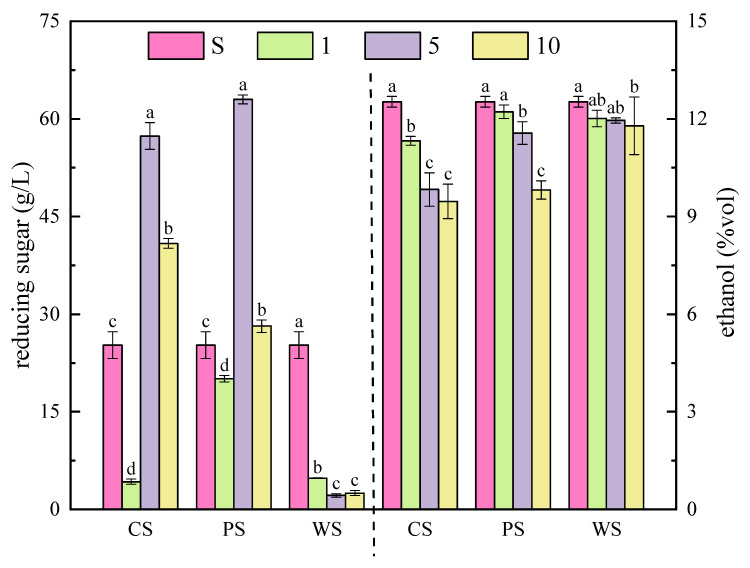
The contents of ethanol and reducing sugar of different ester-producing yeasts with *S. cerevisiae* NCUF309.2 at different ratios on 7 d of simulated fermentation of blueberry wine. (The content of reducing sugar is on the left, and the content of ethanol is on the right. Different letters represent significantly different (*p* < 0.05)).

In the co-fermentation samples, the content of reducing sugar was also consumed rapidly in the early stage of fermentation. Although the change of ethanol content in the co-fermentation samples was similar to that in the sample of S, the ethanol content in the co-fermentation samples was lower than that in the S sample (Figure 3 and Figure 4), indicating that the use of ester-producing yeasts could reduce the ethanol production. In the co-fermentation samples of CS, the consumption of reducing sugar significantly increased in the CS1 sample, and the contents of ethanol decreased with the increases of the *C. glabrata* NCUF308.1 inoculation ratios, which were 11.33%, 9.83%, and 9.46%, respectively. Anyway, the contents of ethanol also decreased with the increases of *P. anomala* NCUF306.1 inoculation ratios, which were 12.22%, 11.56%, and 9.81%, respectively. Belda et al. also found that the final ethanol content was associated with the amount of ester-producing yeast [34]. This could be related to the decrease of the population of *S. cerevisiae* by ester-producing yeast in the samples, which affected the conversion of reducing sugars to ethanol [35]. However, the effects of *W. anomalus* NCUF307.1 on the final ethanol contents were not obvious, which were 12.01%, 11.95%, and 11.79%, respectively. This was accordant with the result of Ye et al., who found that *S. cerevisiae* showed the highest fermentability in the pure fermentation of *S. cerevisiae* with the production of 12.13% ethanol, while the ethanol contents ranged from 11.75% to 12.04% in all mixed fermentations with *S. cerevisiae* and *W. anomalus* [18].

### 3.3. Accumulation of Ester Compounds during the Simulated Fermentation of Blueberry Wine

**Figure 5:**
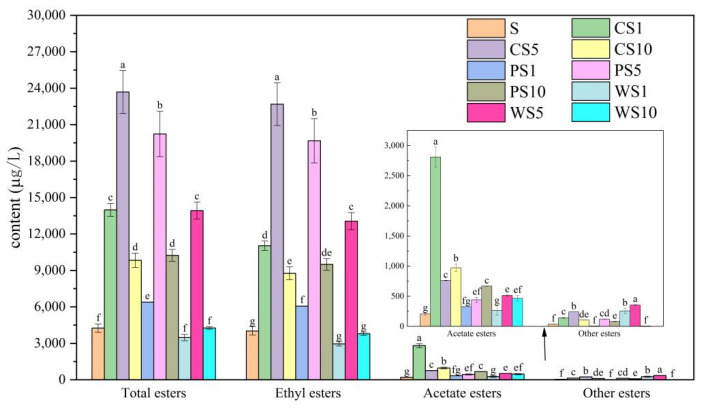
Contents of ester compounds of different ester-producing yeasts, with *S. cerevisiae* NCUF309.2 at different ratios during simulated fermentation of blueberry wine. (Different letters represent significantly different (*p* < 0.05)).

Our results show that, compared with the pure fermentation of *S. cerevisiae* NCUF309.2, the co-fermentation of *S. cerevisiae* NCUF309.2 and *C. glabrata* NCUF308.1 or *P. anomala* NCUF306.1 could significantly increase the contents of ester compounds (*p* < 0.05), which were 3.29, 5.57, 2.34, 1.50, 4.75, and 2.41 times that of S sample, respectively (Figure 5). This could be related to species belonging to the genera of *Candida* and *Pichia*, with a strong capacity to produce ester compounds [36]. Anyway, in the WS samples, the contents of ester compounds significantly increased only in the sample of WS5, which were 3.04 times that of the S sample. The production of ester compounds mainly occurred in the middle and late stages of fermentation. However, in the samples of WS1 and WS10, the population of *W. anomalus* NCUF307.1 in these stages was rare or even absent, resulting in a low content of ester compounds in the samples.

Ester compounds can be further categorized into acetate esters, ethyl esters, and other esters [37]. Both acetate esters and ethyl esters are of key importance in the whole wine flavor, impressing a positive contribution by distinct sensory notes: sweet-fruity, grape-like odor, and sweet-balsamic [38]. In the co-fermentation samples of CS, the contents of acetate esters and ethyl esters both significantly increased, and the content of ethyl esters was higher in the CS5 sample, while the content of acetate esters was higher in the CS1 sample (Figure 5). Additionally, in the co-fermentation samples of PS, the content of ethyl esters was higher in the PS5 sample, while the content of acetate esters was higher in the PS10 sample. These results indicated that the ratio of ester-producing yeast co-fermentation with *S. cerevisiae* had an effect on the formation of ester compounds during fermentation. Lee et al. also found that *W. saturnus* and *S. cerevisiae* could produce a higher content of acetate esters at the ratio of 10:1, while they could produce more ethyl esters at the ratio of 1:1 and 1:10 [39]. In the co-fermentation samples of WS, the contents of acetate esters and ethyl esters both significantly increased in only the WS5 sample. Anyway, the contents of other esters were also significantly increased in the co-fermentation samples (except the samples of PS1 and WS10), and the highest content was in the WS5 sample. 

As for acetate esters, ethyl acetate, phenethyl acetate, and isoamyl acetate were the main acetate esters (Table 1). Additionally, their contents both increased in the co-fermentation samples of CS and PS, while both increased in the co-fermentation samples of WS5 and WS10. Hu et al. also found the contents of ethyl acetate, isoamyl acetate, and phenethyl acetate increased in the co-fermentation samples, compared with those of *S. cerevisiae* pure fermentation [40]. Ethyl acetate could improve the aroma complexity of fruit wine, and phenethyl acetate and isoamyl acetate were recognized as important flavor compounds to contribute to the fruity flavor of fruit wine [41,42]. These results suggested that co-fermentation could improve the acetate contents, especially the contents of ethyl acetate, isoamyl acetate, and phenethyl acetates, compared with those of *S. cerevisiae* pure fermentation, which would be favorable to the flavor of blueberry wine.

As for ethyl esters, medium-chain fatty acid ethyl esters were the main ethyl esters, such as ethyl hexanoate, ethyl octanoate, and ethyl decanoate. Ethyl octanoate has a fruity flavor and is found in many fruits, while ethyl decanoate imparts a fruity and brandy-like flavor to wine [43]. Additionally, the contents of these compounds both significantly increased in the co-fermentation samples of CS and PS, while their contents only significantly increased in the WS5 sample. These results were consistent with the result of a previous study, i.e., that the co-fermentation of *T. delbrueckii* and *S. cerevisiae* had higher contents of ethyl hexanoate, ethyl octanoate, and ethyl decanoate than those of *S. cerevisiae* pure fermentation [44]. In addition, the results also showed that the content of ethyl laurate also increased in the co-fermentation samples of CS and PS. As for other esters, the content of 3-methylbutyl hexanoate increased in the co-fermentation samples of WS, and the content of 3-methylbutyl decanoate increased in the co-fermentation samples of CS. All of these compounds could give the wine more complex flavor characteristics.

These results indicated that different ratios and strains of ester-producing yeast co-fermentation with *S. cerevisiae* could lead to wines with a wide range of ester compounds, which was similar to the results of Comitini et al. [45].

### 3.4. Principal Component Analysis (PCA) of Ester Compounds during the Simulated Fermentation of Blueberry Wine

**Figure 6:**
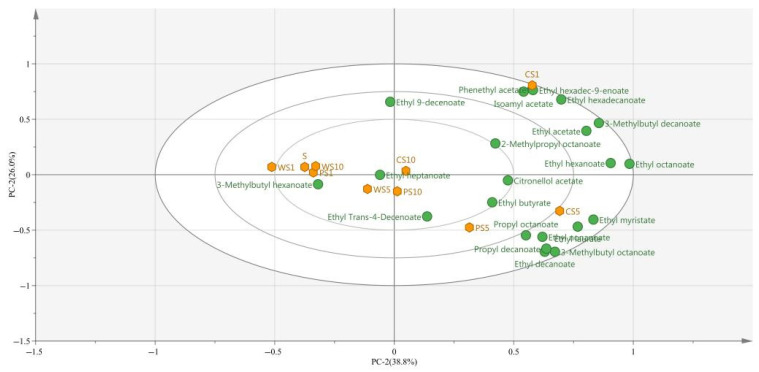
PCA of ester compounds during the simulated fermentation of blueberry wine.

PCA was used to reveal the correlation and separation of ester compounds in different samples (Figure 6). The variance of 64.8% was explained by 29 different ester compounds, and PC1 and PC2 accounted for 38.8% and 26.0% of the variance, respectively. The PCA results showed that the co-fermentation samples of PS1, WS1, and WS10 were close to S sample, suggesting that they had similar ester compound profiles. In these samples, the effects of ester-producing yeast were not obvious, and the characteristics of ester compounds were not prominent. The co-fermentation samples of CS1, CS5, PS5, and WS5 were separated from the S sample and had their own characteristic ester compounds. For example, the sample of CS1 was characterized by phenethyl acetate and isoamyl acetate, the samples of CS5 and PS5 were characterized by propyl octanoate and ethyl decanoate, and the sample of WS5 was characterized by 3-methylbutyl hexanoate, which provided the potential for creating a distinctive flavor of blueberry wine.

### 3.5. Volatile Odor Active Ester Compounds during the Simulated Fermentation of Blueberry Wine

The odor activity value (OAV) is a common index used to evaluate the contribution of volatile compounds of wine to the actual flavor, which can be calculated by the ratio of the content of the volatile compound to its odor threshold [46]. It is generally believed that an OAV greater than 1 indicates that it contributes to the odor, and a larger OAV indicates a greater individual contribution of the compound [47]. The ester compounds with OAV greater than 1 are shown in Table 2. In the S sample, the OAVs of phenethyl acetate and isoamyl acetate were lower than 1; however, the OAVs of phenethyl acetate and isoamyl acetate were greater than 1 in the CS1, CS5, CS10, PS5, PS10, WS5, and WS10 samples, indicating that co-fermentation directly promoted the contribution of phenethyl acetate and isoamyl acetate to the fruity and floral flavors. Although the content of isoamyl acetate was lower than that of phenethyl acetate, its threshold was lower, leading to a higher OAV than phenethyl acetate. The OAV of phenethyl acetate and isoamyl acetate were both the highest in the sample of CS1, which was 9.68 and 32.33 times that of the S sample, suggesting that they could contribute to more floral and fruity flavors in the sample of CS1. 

As for ethyl esters, ethyl octanoate, with a high content and low threshold (5 μg/L), had the greatest influence on flavor of fruit wine and could give wine flavors of pineapple, pear, and floral [48]. The OAV of ethyl hexanoate with the same low threshold (5 μg/L) was second only to ethyl octanoate and could give wines with green apple and purple apple flavors. The OAVs of ethyl octanoate and ethyl hexanoate were the highest in the CS1 sample, which were 1350.05 and 116.43. In addition, ethyl decanoate also had significant effects on the overall flavor and could directly promote the fruity and floral flavors of samples [49]. Anyway, the OAV of ethyl laurate was greater than 1 in only the samples of CS1, CS5, PS1, and PS5, thus directly giving these samples sweet, waxiness, and floral flavors.

## 4. Conclusions

In this study, *S. cerevisiae* NCUF309.2 was fermented with three different ester-producing yeasts (*C. glabrata* NCUF308.1, *P. anomala* NCUF306.1, and *W. anomalus* NCUF307.1) under different inoculation ratios to simulate the co-fermentation of blueberry wine. The growth of the yeasts, fermentation characteristics, and the accumulation of ester compounds during the fermentation were studied. The results show that, compared with the S sample, the presence of *P. anomala* NCUF306.1 and *C. glabrata* NCUF308.1 could affect the growth of *S. cerevisiae* NCUF309.2 and decrease the population of *S. cerevisiae* NCUF309.2, leading to a decrease in the content of ethanol. However, the growth of *S. cerevisiae* NCUF309.2 was not affected in the sample of WS5, and the presence of *W. anomalus* NCUF307.1 had little effect on ethanol content. The results also showed that, compared with the S sample, the co-fermentation of *S. cerevisiae* NCUF309.2 with ester-producing yeast could produce wine with distinct flavor characteristics (Figure 6), especially in the samples of CS1, PS5, and WS5. Additionally, the contents of ester compounds in these samples were significantly increased. Among them, the CS1 sample had the highest contents of odor active compounds, including phenethyl acetate, isoamyl acetate, ethyl octanoate, and ethyl hexanoate, which could directly endow fruit wine with more floral and fruity flavor, compared with other samples. In conclusion, the co-fermentation of *C. glabrata* NCUF308.1 and *S. cerevisiae* NCUF309.2 at the ratio of 1:1 was a feasible method for improving the aroma diversity of blueberry wine and meet the requirements of consumers.

## Figures and Tables

**Table 1:** Type and content of ester compounds identified in different ester-producing yeasts with *S. cerevisiae* NCUF309.2 at different ratios during simulated fermentation of blueberry wine.

Compounds	Content (μg/L)									
	S	CS1	CS5	CS10	PS1	PS5	PS10	WS1	WS5	WS10
**Acetate esters**										
Ethyl acetate	8.28 ± 1.24 ^f^	326.48 ± 11.65 ^a^	302.71 ± 1.75 ^b^	107.51 ± 2.05 ^c^	15.09 ± 0.1 ^f^	23.68 ± 1.12 ^e^	11.43 ± 1.50 ^f^	14.17 ± 3.33 ^f^	33.76 ± 5.63 ^d^	36.49 ± 6.64 ^d^
Isoamyl acetate	24.93 ± 1.78 ^f^	805.01 ± 39.97 ^a^	126.93 ± 25.73 ^d^	456.2 ± 30.69 ^b^	73.34 ± 2.18 ^e^	79.11 ± 5.12 ^e^	186.68 ± 2.61 ^c^	14.51 ± 3.82 ^f^	105.49 ± 10.04 ^de^	173.91 ± 15.36 ^c^
Phenethyl acetate	170.96 ± 20.35 ^c^	1671.16 ± 256.88 ^a^	322.26 ± 35.56 ^bc^	501.54 ± 81.34 ^b^	238.74 ± 1.45 ^c^	335.12 ± 68.01 ^bc^	455.92 ± 0.56 ^b^	214.89 ± 97.86 ^c^	348.59 ± 14.23 ^bc^	254.87 ± 60.73 ^c^
Citronellyl acetate	n.d	7.61 ± 1.45 ^b^	8.53 ± 3.37 ^b^	6.73 ± 1.38 ^b^	n.d	2.44 ± 0.17 ^c^	13.85 ± 0.97 ^a^	n.d	11.68 ± 1.49 ^a^	n.d
Decyl acetate	n.d	n.d	n.d	n.d	n.d	n.d	n.d	n.d	13.11 ± 2.65	n.d
**Ethyl esters**										
Ethyl butyrate	n.d	3.44 ± 0.4 ^d^	4.85 ± 0.4 ^bc^	5.84 ± 0.44 ^ab^	4.12 ± 0.06 ^cd^	6.74 ± 0.82 ^a^	5.91 ± 0.36 ^ab^	n.d	n.d	6.73 ± 1.25 ^a^
Ethyl hexanoate	62.41 ± 7.664 ^e^	582.13 ± 76.57 ^a^	520.42 ± 73.34 ^ab^	407.55 ± 48.56 ^bc^	192.75 ± 1.38 ^d^	368.06 ± 62.20 ^c^	315.04 ± 160.49 ^cd^	33.05 ± 1.89 ^e^	417.24 ± 61.95 ^bc^	195.36 ± 0.99 ^d^
Ethyl heptanoate	3.97 ± 1.3 ^d^	5.7 ± 1.45 ^cd^	3.23 ± 0.38 ^d^	23.80 ± 8.34 ^b^	4.56 ± 0.01 ^d^	n.d	38.36 ± 5.99 ^a^	12.00 ± 0.56 ^c^	n.d	2.83 ± 0.45 ^d^
Ethyl octanoate	1763.28 ± 76.86 ^e^	6750.23 ± 481.42 ^a^	6347.14 ± 42.91 ^a^	3996.98 ± 506.14 ^c^	1794.26 ± 249.20 ^e^	5085.02 ± 689.79 ^b^	3028.91 ± 525.23 ^d^	1427.26 ± 254.62 ^e^	3376.21 ± 498.56 ^cd^	1859.77 ± 204.61 ^e^
Ethyl nonanoate	5.62 ± 0.68 ^b^	1.96 ± 0.17 ^c^	18.18 ± 0.45 ^a^	17.35 ± 1.19 ^a^	n.d	16.93 ± 1.55 ^a^	4.92 ± 0.29 ^b^	n.d	5.21 ± 0.45 ^b^	n.d
Ethyl 9-decenoate	1567.7 ± 153.76 ^a^	1462.57 ± 46.68 ^a^	1023.39 ± 236.30 ^b^	732.31 ± 125.88 ^c^	567.44 ± 110.42 ^d^	27.91 ± 2.77 ^e^	45.82 ± 7.6 ^e^	1054.97 ± 64.15 ^b^	560.53 ± 71.10 ^d^	806.55 ± 146.26 ^c^
Ethyl decanoate	484.76 ± 357.86 ^e^	1006.54 ± 73.59 ^de^	12156.92 ± 2556.84 ^a^	3200.49 ± 202.31 ^c^	2493.39 ± 456.48 ^cd^	10439.39 ± 2087.73 ^a^	2612.85 ± 238.33 ^cd^	320.28 ± 23.88 ^e^	7693.33 ± 519.71 ^b^	811.22 ± 99.28 ^de^
Ethyl trans-4-decenoate	12.80 ± 1.94 ^c^	n.d	n.d	n.d	n.d	1587.57 ± 166.99 ^b^	3158.02 ± 48.04 ^a^	n.d	n.d	n.d
Ethyl E-11-hexadecenoate	n.d	18.39 ± 0.55	n.d	n.d	n.d	n.d	n.d	n.d	n.d	7.51 ± 0.87
Ethyl laurate	88.08 ± 12.05 ^de^	843.53 ± 37.61 ^c^	2490.92 ± 244.30 ^a^	312.81 ± 21.5 ^d^	822.51 ± 143.81 ^c^	2064.04 ± 320.08 ^b^	228.19 ± 0.78 ^de^	35.99 ± 4.19 ^e^	n.d	67.9 ± 3.68 ^de^
Ethyl undecanoate	n.d	n.d	n.d	n.d	128.59 ± 23.89	n.d	n.d	n.d	n.d	n.d
Ethyl myristate	2.37 ± 0.23 ^c^	6.29 ± 0.46 ^bc^	24.57 ± 0.45 ^a^	3.90 ± 0.67 ^c^	n.d	11.15 ± 8.79 ^b^	5.01 ± 0.18 ^c^	n.d	3.74 ± 0.3 ^c^	n.d
Ethyl hexadec-9-enoate	16.68 ± 1.68 ^d^	199.43 ± 12.59 ^a^	46.44 ± 11.86 ^b^	40.30 ± 3.56 ^b^	44.74 ± 3.71 ^b^	38.57 ± 19.42 ^b^	44.79 ± 5.38 ^b^	22.69 ± 2.09 ^cd^	10.93 ± 3.28 ^d^	33.44 ± 0.27 ^bc^
Ethyl hexadecanoate	6.93 ± 0.95 ^d^	167.22 ± 9.24 ^a^	51.05 ± 11.19 ^b^	19.16 ± 3.03 ^c^	7.43 ± 0.63 ^d^	28.47 ± 7.11 ^c^	6.71 ± 3.52 ^d^	5.27 ± 0.29 ^d^	18.74 ± 3.79 ^c^	7.67 ± 0.88 ^d^
Ethyl oleate	n.d	6.75 ± 1.21	n.d	n.d	n.d	n.d	n.d	n.d	2.57 ± 0.43	n.d
**Other esters**										
Isobutyl caproate	n.d	n.d	n.d	1.23 ± 0.35	n.d	n.d	n.d	n.d	n.d	n.d
3-methylbutyl hexanoate	n.d	n.d	7.98 ± 0.77 ^c^	10.26 ± 2.53 ^c^	1.88 ± 0.19 ^c^	n.d	12.09 ± 2.22 ^c^	250.94 ± 43.32 ^b^	298.38 ± 26.12 ^a^	0.95 ± 0.43 ^c^
Propyl octanoate	1.89 ± 0.21 ^c^	2.25 ± 0.39 ^c^	10.69 ± 1.75 ^b^	n.d	n.d	13.46 ± 2.64 ^ab^	14.53 ± 2.56 ^a^	n.d	n.d	n.d
2-methylpropyl octanoate	10.50 ± 1.34 ^c^	14.85 ± 1.62 ^b^	5.92 ± 1.18 ^de^	8.50 ± 1.81 ^cd^	n.d	4.48 ± 0.86 ^e^	19.38 ± 2.99 ^a^	n.d	n.d	n.d
3-methylbutyl octanoate	22.12 ± 2.48 ^d^	7.7 ± 0.79 ^e^	89.52 ± 13.69 ^a^	39.19 ± 2.01 ^c^	3.87 ± 0.36 ^e^	66.99 ± 10.39 ^b^	36.01 ± 3.46 ^c^	5.67 ± 0.62 ^e^	34.11 ± 7.83 ^c^	4.52 ± 2.04 ^e^
Octyl 2-methylbutyrate	n.d	n.d	19.22 ± 1.37	5.24 ± 0.45	n.d	n.d	n.d	n.d	n.d	n.d
Propyl decanoate	n.d	n.d	18.20 ± 0.36 ^a^	2.26 ± 0.67 ^d^	n.d	15.92 ± 1.42 ^b^	n.d	n.d	8.02 ± 0.68 ^c^	n.d
Isobutyl decanoate	n.d	n.d	7.42 ± 2.16	4.24 ± 0.54	n.d	n.d	n.d	n.d	n.d	n.d
3-methylbutyl decanoate	1.97 ± 0.16 ^d^	117.24 ± 23.68 ^a^	81.58 ± 13.34 ^b^	33.6 ± 2.42 ^c^	n.d	20.12 ± 4.57 ^c^	n.d	n.d	11.95 ± 1.72 ^d^	1.23 ± 0.09 ^d^

Note: n.d, undetected (different letters represent significantly different (*p* < 0.05)).

**Table 2:** Volatile odor active compounds (OAV > 1) in fermentation samples during the simulated fermentation of fruit wine.

Compounds	Threshold (μg/L)	OAV									
		S	CS1	CS5	CS10	PS1	PS5	PS10	WS1	WS5	WS10
Phenethyl acetate	250	0.68 ± 0.06 ^c^	6.68 ± 0.59 ^a^	1.29 ± 0.08 ^bc^	2.01 ± 0.19 ^b^	0.95 ± 0.00 ^c^	1.34 ± 0.19 ^bc^	1.82 ± 0.00 ^b^	0.86 ± 0.39 ^c^	1.39 ± 0.04 ^bc^	1.02 ± 0.17 ^c^
Isoamyl acetate	30	0.83 ± 0.04 ^f^	26.83 ± 0.77 ^a^	4.23 ± 0.50 ^d^	15.21 ± 0.59 ^b^	2.44 ± 0.05 ^e^	2.64 ± 0.12 ^e^	6.22 ± 0.06 ^c^	0.48 ± 0.13 ^f^	3.52 ± 0.24 ^de^	5.80 ± 0.36 ^c^
Ethyl octanoate	5	352.66 ± 10.87 ^e^	1350.05 ± 55.59 ^a^	1269.43 ± 4.95 ^a^	799.4 ± 58.44 ^c^	358.85 ± 35.24 ^e^	1017.00 ± 97.55 ^b^	605.78 ± 74.28 ^d^	285.45 ± 50.92 ^e^	675.24 ± 70.51 ^cd^	371.95 ± 28.94 ^e^
Ethyl hexanoate	5	12.48 ± 1.08 ^e^	116.43 ± 8.84 ^a^	104.08 ± 8.47 ^ab^	81.51 ± 5.61 ^bc^	38.55 ± 0.20 ^d^	73.61 ± 8.80 ^bc^	63.00 ± 22.70 ^cd^	6.61 ± 0.38 ^e^	83.45 ± 8.76 ^bc^	39.07 ± 0.14 ^d^
Ethyl 9-decenoate	100	15.68 ± 1.09 ^a^	14.63 ± 0.27 ^a^	10.23 ± 1.36 ^b^	7.32 ± 0.73 ^c^	5.67 ± 0.78 ^d^	0.28 ± 0.02 ^e^	0.46 ± 0.05 ^e^	10.55 ± 0.58 ^b^	5.61 ± 0.50 ^d^	8.07 ± 1.03 ^c^
Ethyl decanoate	200	2.42 ± 1.27 ^e^	5.03 ± 0.21 ^de^	60.78 ± 7.38 ^a^	16.00 ± 0.58 ^c^	12.47 ± 1.61 ^cd^	52.20 ± 7.38 ^a^	13.06 ± 0.84 ^cd^	1.60 ± 0.12 ^e^	38.47 ± 1.84 ^b^	4.06 ± 0.35 ^de^
Ethyl laurate	800	0.11 ± 0.01 ^de^	1.05 ± 0.03 ^c^	3.11 ± 0.18 ^a^	0.39 ± 0.02 ^d^	1.03 ± 0.13 ^c^	2.58 ± 0.28 ^b^	0.29 ± 0.00 ^de^	0.04 ± 0.01 ^e^	n.d	0.08 ± 0.00 ^de^

Note: n.d, undetected (different letters represent significantly different (*p* < 0.05)).

## Data Availability

The datasets generated for this study are available on request to the corresponding author.

## Supplementary Materials

The following supporting information can be downloaded at: https://www.mdpi.com/article/10.3390/foods11223655/s1, Table S1: The population of *S. cerevisiae* NCUF309.2 during the simulated fermentation of blueberry wine.
