# Supplementary material for: Increase the Content of Ester Compounds in Blueberry Wine Fermentation with the Ester-Producing Yeast: Candida glabrata, Pichia anomala, and Wickerhamomyces anomalus
Source: Foods. 2022 Nov 16;11(22):3655. doi: 10.3390/foods11223655 (PMC9689967; doi:10.3390/foods11223655)
Supplement: Supplementary file 1 [file foods-11-03655-s001.zip › Supplementary Table S1.pdf]

Table S1 The population of *S. cerevisiae* NCUF309.2 during the simulated fermentation of blueberry wine.

Note: n.d, undetected (different letters represented significantly different ($p<0.05$)).

day	population ($\times 10^7$ CFU /mL)											
	CS				PS				WS			
	**S**	**CS1**	**CS5**	**CS10**	**S**	**PS1**	**PS5**	**PS10**	**S**	**WS1**	**WS5**	**WS10**
0	0.01±0.00^{a}	0.01±0.00^{a}	0.01±0.00^{a}	0.01±0.00^{a}	0.01±0.00^{a}	0.01±0.00^{a}	0.01±0.00^{a}	0.01±0.00^{a}	0.01±0.00^{a}	0.01±0.00^{a}	0.01±0.00^{a}	0.01±0.00^{a}
1	0.20±0.09^{b}	3.33±1.15^{a}	4.33±1.53^{a}	0.67±0.58^{b}	0.20±0.09^{b}	3.67±1.53^{a}	4.67±1.53^{a}	1.00±0.00^{b}	0.20±0.09^{d}	5.67±1.53^{b}	19.00±1.00^{a}	3.00±1.73^{c}
2	3.00±0.26^{b}	44.33±5.03^{a}	5.67±0.58^{b}	3.00±1.00^{b}	3.00±0.26^{c}	6.33±1.15^{b}	11.00±1.00^{a}	1.67±0.58^{c}	3.00±0.26^{c}	15.67±2.52^{b}	31.67±6.03^{a}	6.00±1.73^{c}
3	7.63±1.00^{b}	38.00±6.56^{a}	10.00±2.65^{b}	4.33±1.15^{b}	7.63±1.00^{c}	17.67±2.52^{a}	12.33±2.08^{b}	4.33±1.53^{c}	7.63±1.00^{c}	15.33±2.52^{b}	37.33±6.51^{a}	10.67±1.53^{bc}
4	22.05±3.54^{b}	36.00±5.29^{a}	17.00±3.61^{b}	5.67±1.15^{c}	22.5±3.54^{b}	30.33±3.51^{a}	13.33±2.52^{c}	5.67±1.53^{d}	22.5±3.54^{ab}	20.67±3.06^{b}	28.67±5.69^{a}	9.00±2.00^{c}
5	26.67±1.53^{a}	15.00±2.65^{c}	21.00±4.00^{b}	7.33±1.53^{d}	26.67±1.53^{a}	25.33±1.53^{a}	9.00±3.00^{b}	7.67±1.15^{b}	26.67±1.53^{a}	17.00±2.00^{b}	22.67±3.79^{a}	9.67±3.06^{c}
6	34.33±4.51^{a}	16.33±2.52^{b}	14.67±3.51^{b}	15.33±3.06^{b}	34.33±4.51^{a}	28.67±6.81^{a}	7.33±3.51^{b}	13.33±2.52^{b}	34.33±4.51^{a}	12.67±4.16^{c}	23.33±5.13^{b}	17.00±2.65^{bc}
7	28.67±2.52^{a}	13±4.58^{b}	15.00±4.00^{b}	23.00±4.00^{a}	28.67±2.52^{a}	20.67±3.51^{ab}	7.67±2.52^{c}	20.00±7.00^{b}	28.67±2.52^{a}	13.33±2.08^{c}	18.00±4.36^{bc}	19.67±3.06^{b}
